# Maternal Satisfaction and Childbirth Experience Following Vaginal Delivery Versus Cesarean Section: A Systematic Review

**DOI:** 10.7759/cureus.110624

**Published:** 2026-06-10

**Authors:** Majed Almutairi

**Affiliations:** 1 Department of Obstetrics and Gynecology, Faculty of Medicine, King Abdulaziz University, Rabigh, SAU

**Keywords:** cesarean section, maternal care satisfaction, mode of delivery, patient experience, patient’s satisfaction, systematic review, vaginal delivery

## Abstract

Childbirth is a crucial experience that impacts women's lives, and the choice between vaginal delivery and cesarean section (C-section) is a critical decision in obstetrics. Maternal satisfaction is influenced by multiple factors since the childbirth experience is a composite of physical, emotional, and social components. Understanding patient evaluation is crucial for providing patient-centered care and improving maternity and neonatal care services. This systematic review aimed to compare patient satisfaction between vaginal and cesarean delivery patients and identify influencing factors.

We performed a thorough search of databases for studies published between 2000 and 2024 on patient-reported satisfaction with vaginal delivery vs. C-section interventions. Eligible studies were assessed for methodological quality and relevance.

The findings indicated that most women were satisfied with their delivery experience, with vaginal delivery leading to higher satisfaction than C-sections. Factors influencing satisfaction include maternal education, domicile, planned delivery care, healthcare professional gender, complications, partners' education, pain control measures, Apgar scores, and injuries. However, satisfaction levels were not significantly different across other maternal demographic factors or pregnancy-related characteristics. Certain features, such as planned pregnancy and excellent prenatal care, improved satisfaction with both vaginal and cesarean deliveries. The presence of a supporting companion during birth significantly boosted satisfaction levels, especially in primary care settings. Inadequate communication is associated with decreased maternal satisfaction.

Therefore, healthcare practitioners should prioritize patient-centered care, good communication, and support. Targeted interventions are recommended, considering factors that influence the delivery of maternal and child care services.

## Introduction and background

Childbirth is a transformative phenomenon that immensely affects women’s lives temporarily or permanently, and the mode of delivery is a key determinant of satisfaction and a better experience [1]. The choice between vaginal delivery and cesarean section (C-section) is among the most critical decisions in obstetrics, not only affecting maternal health but also influencing neonatal outcomes [1,2]. Studies have found that maternal satisfaction is multifaceted and influenced by different factors regarding the delivery process, indicating differences between the satisfaction of women who have had C-sections and vaginal deliveries. Maternal education, residing in an urban or rural area, the gender of her healthcare provider, and the type of healthcare facility were found to significantly affect the satisfaction levels of women regarding either type of delivery [3,4]. Moreover, obstetric procedures and complications have been established as significant predictors of women's satisfaction with childbirth. Emergency C-sections, postpartum hemorrhage, and Apgar ratings below 7 at five minutes were identified as risk factors for dissatisfaction with childbirth, underscoring the importance of medical interventions on maternal experience [5]. Other non-invasive processes, such as written or verbal consent signed by patients, also significantly affect patient satisfaction. It was found that good communication and choice processes played a vital role in patient satisfaction and improved women's experiences [6]. Social determinants like planned pregnancy, spousal support during delivery, emotional support, antenatal care program sufficiency, pain management, and maternal engagement in decision-making were also found to be associated with maternal satisfaction with vaginal delivery or C-section [7]. In Geneva, people viewed the two delivery methods differently, associating C-sections with negativity. Similarly, Iranian and UK studies found that vaginal delivery was perceived as a safer mode than C-section. C-section was perceived as being associated with more complications and poor maternal adjustment post-delivery [2,8], explaining why research evidence from different settings worldwide showed that mothers were more satisfied with vaginal delivery than C-section delivery [4,5,8,9]. One study reported a higher satisfaction in women who had a C-section, and a low level of satisfaction in women who had a normal birth in the early postpartum period [7], contrasting other studies’ findings. However, Dinc et al. reported no significant difference in mean satisfaction scores between those who had vaginal delivery and those who had a C-section based on both socio-demographic factors and care service-related factors (p>0.05) [10]. 

The childbirth experience is a complex phenomenon shaped by physical, emotional, psychological, and social factors that collectively influence maternal satisfaction. Although numerous studies have compared satisfaction between vaginal delivery and C-section, their findings remain heterogeneous and sometimes contradictory. While several studies report higher satisfaction following vaginal delivery, others have demonstrated comparable or even higher satisfaction among women undergoing C-section, particularly when the procedure is planned and accompanied by adequate counseling and support. This variability makes it difficult for clinicians and policymakers to draw definitive conclusions regarding the relationship between mode of delivery and maternal satisfaction. Therefore, a systematic synthesis of the available evidence is needed to provide a comprehensive overview of maternal satisfaction across delivery modes and identify the factors that influence women's childbirth experiences. The findings may assist healthcare providers in improving patient-centered maternity care and enhancing the quality of obstetric services. Accordingly, this systematic review aimed to compare maternal satisfaction between vaginal delivery and C-section and to identify factors associated with satisfaction across different delivery modes.

## Review

Methods

This was a systematic review conducted following the Preferred Reporting Items for Systematic Reviews and Meta-Analyses (PRISMA) guidelines for reporting, and this review answered this search question: “What is the satisfaction between patients who give birth by normal vaginal delivery and those who underwent C-section?" This review was not prospectively registered in the International Prospective Register of Systematic Reviews (PROSPERO) or any other systematic review registry.

Search Strategy

A comprehensive search was performed in electronic databases, including Cochrane, Medline/PubMed, Google Scholar, Web of Science, Scopus, and Excerpta Medica Database (EMBASE). The literature search was conducted from January 2025 to March 2025, and the final search was completed on 29 March 2025. The search strategy included a combination of medical subject headings (MeSH) terms and keywords related to childbirth, delivery mode, and patient satisfaction: “Patient Satisfaction," Childbirth Satisfaction," “Vaginal Delivery Satisfaction," “Cesarean Section Satisfaction," “Healthcare Provider-Patient Relations," “Cesarean Section," “Mode of Delivery,” "Obstetric," and “Birth Experience."

The complete search strategies used for each database are provided in Appendix 1. An example PubMed search strategy was: ("Patient Satisfaction"[MeSH] OR satisfaction OR childbirth experience OR birth experience) AND ("Cesarean Section"[MeSH] OR cesarean section OR caesarean section) AND ("Vaginal Delivery"[MeSH] OR vaginal birth OR normal delivery). To optimize the search outcomes, we combined the search terms using Boolean operators (AND, OR) for the keywords and MeSH terms mentioned above. We also manually searched the references of relevant articles to identify additional new studies that could have been missed during the original search.

Our search strategy followed the Population, Intervention, Comparison, and Outcome (PICO) structure as follows: we targeted studies conducted on adult women aged above 18 who gave birth to at least one baby (Population) and who underwent a C-section (Intervention), and those who gave birth by vaginal delivery (Comparison). The primary outcome (Outcome) was maternal satisfaction with childbirth or delivery care. Secondary outcomes included childbirth experience, satisfaction with informed consent, willingness to choose the same delivery mode in future pregnancies, perceived control during childbirth, psychological adjustment following birth, and factors associated with satisfaction.

Study Selection

The author screened all retrieved articles against predefined inclusion and exclusion criteria. Studies were included if they met the following criteria: original research articles, such as controlled trials, prospective cohort studies, retrospective studies, case-control studies, and other observational studies published in peer-reviewed journals; studies comparing maternal satisfaction, childbirth experience, or patient-reported outcomes between vaginal delivery, operative vaginal delivery, elective C-section, emergency C-section, vaginal birth after cesarean (VBAC), or C-section after previous vaginal birth; studies reporting quantitative measures of patient satisfaction using validated instruments or self-reported outcomes; studies published in the English language; published in the last 24 years, from 2000 to 2024; and studies involving women aged 18 years or older who gave birth in the past. 

Case reports, review articles, conference abstracts, theses, studies published before the year 2000, those published in non-peer-reviewed journals, non-English language publications, and those that did not evaluate patient outcomes of our interest were excluded.

**Figure 1:**
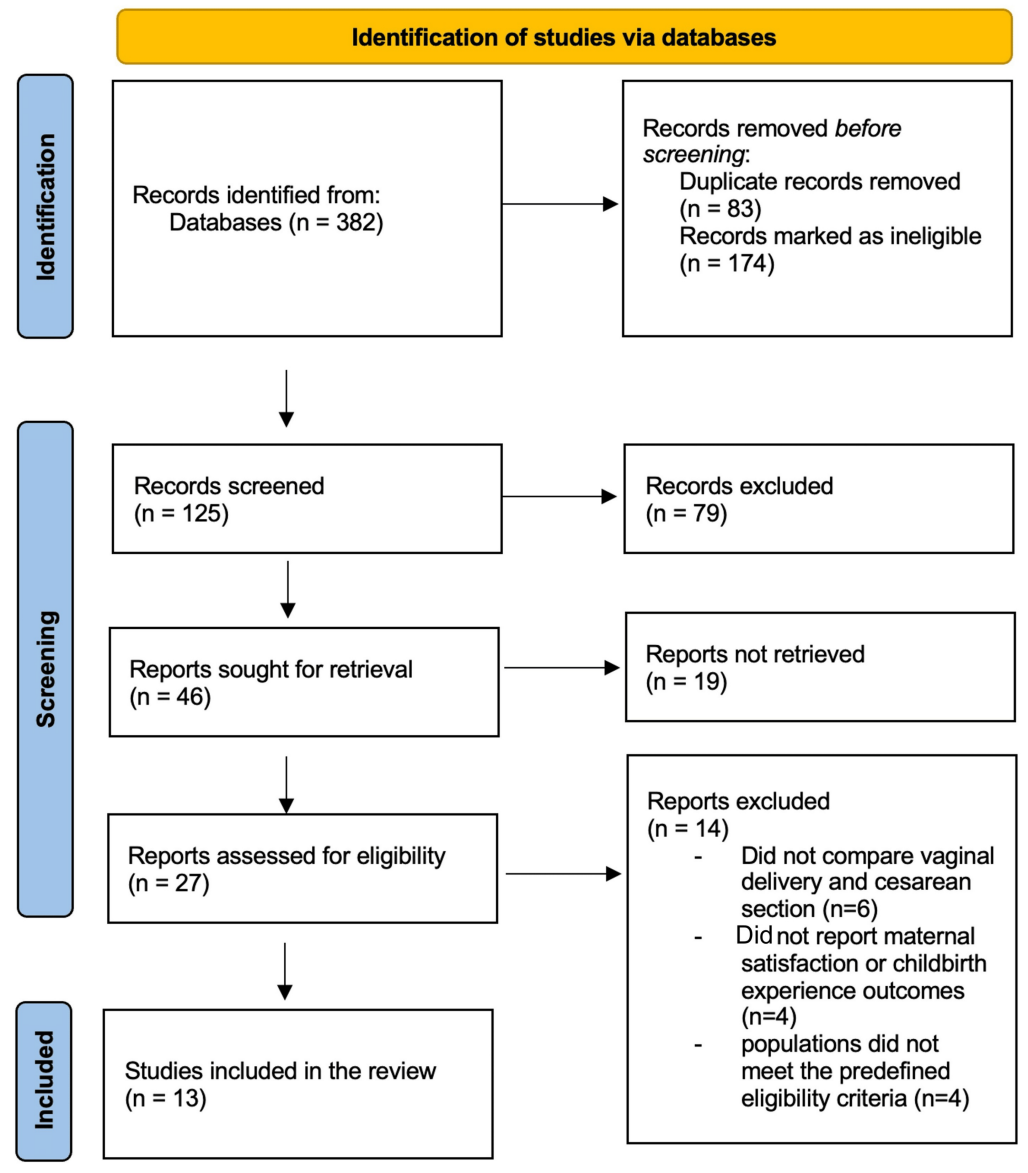
PRISMA flow chart illustrating the process of selecting studies PRISMA: Preferred Reporting Items for Systematic reviews and Meta-Analyses

We retrieved and reviewed full-text publications of possibly relevant articles, evaluating them for inclusion. Any uncertainties regarding study eligibility were resolved through repeated assessment of the predefined inclusion and exclusion criteria. Figure 1 shows the procedure for choosing the included studies.

Data Extraction and Quality Assessment

Data from eligible studies were extracted by the author using a standardized data extraction form. Extracted information included author name, publication year, country, study design, sample size, delivery mode, satisfaction measurement tool, and key findings. To ensure consistency, extracted information was reviewed repeatedly against the original articles before inclusion in the synthesis.

Methodological quality was assessed by the author using appraisal tools appropriate for each study design. The Cochrane Risk of Bias (RoB) Tool [11] was used for randomized studies, the Newcastle-Ottawa Scale (NOS) [12] for observational studies, and the qualitative studies were assessed using the Critical Appraisal Skills Programme (CASP) Qualitative Checklist [13]. The results of each assessment are presented separately according to study design and appraisal instrument (Appendices 2-4).

Narrative Synthesis

A narrative synthesis of findings was conducted to summarize the key findings of the included studies, and data were presented based on two themes: Satisfaction and factors associated with satisfaction regarding vaginal vs cesarean delivery. 

Results

A total of 382 records were identified through database searching. After the removal of 83 duplicate records, 299 unique records remained. Of these, 174 records were excluded during preliminary screening because they were clearly unrelated to childbirth satisfaction, involved non-human subjects, represented review articles, conference abstracts, or editorials, or did not address delivery mode. The remaining 125 records underwent title and abstract screening, of which 79 were excluded. Forty-six full-text articles were sought for retrieval; 19 could not be retrieved. Twenty-seven articles underwent full-text assessment, and 14 were excluded because they did not compare delivery modes, did not report maternal satisfaction outcomes, involved inappropriate populations, or were review articles. Thirteen studies met the inclusion criteria and were included in the final review (Table 1).

**Table 1:** Characteristics of the included articles ANC: antenatal care; aOR: adjusted odds ratio; CI: confidence interval; CS: cesarean section; CSAVD: cesarean section after previous vaginal delivery; HIV: human immunodeficiency virus; OR: odds ratio; OVD: operative vaginal delivery; VBAC: vaginal birth after cesarean section. All statistical estimates, including p-values and confidence intervals, were extracted from the original studies

Authors	Year	Country	Study Design	Study Population	Total Sample Size	Exposure Group (n)	Comparison Group (n)	Satisfaction Measurement Tool	Key Relevant Characteristics	Main Findings
Dunn et al. [14]	2005	Ireland	Prospective questionnaire study	Women with a previous delivery history	140	VBAC (70)	Cesarean after previous vaginal birth (70)	Postnatal questionnaire	Previous delivery history; early postpartum assessment	VBAC women reported higher satisfaction, less discomfort, and greater preparedness
Karoni et al. [4]	2020	Ethiopia	Comparative cross-sectional	Postpartum mothers in health facilities	444	Vaginal delivery (222)	Cesarean delivery (222)	Maternal satisfaction questionnaire	Public and private facilities; urban/rural residence	Satisfaction is higher among vaginal delivery mothers (65.6% vs 57.2%)
Yilmaz et al. [10]	2020	Turkey	Cross-sectional	Postpartum women	351	Vaginal delivery	Cesarean delivery	Birth satisfaction scale	Evaluated socio-demographic and obstetric factors	No significant difference in satisfaction between groups
Semiha Aydin et al. [7]	2017	Turkey	Cross-sectional	Women in the postpartum period	300	Vaginal delivery	Cesarean delivery	Maternal satisfaction scale	Examined partner characteristics and prenatal care	Cesarean delivery is associated with higher satisfaction (81.3% vs 70.4%)
Levy et al. [6]	2022	Canada	Cross-sectional	Women undergoing operative deliveries	224	Elective and emergency cesarean section	Operative vaginal delivery	Informed consent satisfaction questionnaire	Focused on the consent process and recall	Higher satisfaction among elective and emergency CS than operative vaginal delivery
Alderdice et al. [8]	2019	United Kingdom	Cross-sectional population survey	Postpartum mothers	4,578	Planned and unplanned cesarean section	Vaginal birth	Standardized postnatal adjustment questionnaire	Psychological mediators evaluated	Unplanned CS is associated with poorer maternal adjustment and lower satisfaction
Porter et al. [15]	2007	United Kingdom	Qualitative study	Women reporting dissatisfaction after CS	1,297 survey respondents	Cesarean section	Not applicable	Open-ended qualitative responses	Explored causes of dissatisfaction	Poor communication and anxiety were major contributors to dissatisfaction
Falk et al. [5]	2019	Sweden	Population-based retrospective cohort	Women delivering in Swedish hospitals	16,775	Emergency CS and obstetric interventions	Spontaneous vaginal delivery	Childbirth experience questionnaire	Registry-based cohort	Emergency CS is strongly associated with dissatisfaction
Zakerihamidi et al. [2]	2015	Iran	Qualitative ethnographic study	Pregnant and postpartum women	45	Cesarean section	Vaginal delivery	Semi-structured interviews	Explored perceptions of delivery modes	Vaginal birth is viewed positively; CS is viewed as pain-free but associated with future risks
Schindl et al. [16]	2003	Austria	Prospective trial	Pregnant women	1,050	Elective cesarean section (147)	Planned vaginal birth (903)	Birth experience questionnaire	Compared birth experience and complications	Elective CS is associated with a better birth experience than emergency CS
Guittier et al. [1]	2014	Switzerland	Qualitative study	First-time mothers	40	Cesarean section	Vaginal delivery	In-depth interviews	Explored perceptions and birth experience	Delivery mode influenced perceived control and emotions
Mohammed et al. [17]	2015	Egypt	Cross-sectional	Women with previous births	60	VBAC (30)	Cesarean after vaginal birth (30)	Structured satisfaction questionnaire	Focused on repeat birth experiences	Satisfaction is significantly higher in the VBAC group
Costa et al. [18]	2023	Brazil	Cohort study	Women hospitalized for childbirth	23,894	Cesarean section	Vaginal delivery	Birth in Brazil satisfaction scale	National multicenter study	No significant difference after adjustment

Quality Assessment of Included Studies

The methodological quality of the included studies was assessed using appraisal tools appropriate for each study design. Observational studies were evaluated using the NOS [12], qualitative studies were assessed using the CASP Qualitative Checklist [13], and the prospective trial was evaluated using the RoB 2 tool [11]. Most observational studies were of high methodological quality, with NOS scores ranging from seven to nine stars. The principal limitations identified were limited adjustment for confounding variables, single-center recruitment, and potential recall bias in studies relying on self-reported satisfaction measures. All qualitative studies demonstrated good methodological rigor, although researcher reflexivity and transferability were not consistently addressed. The prospective trial showed low risk of bias across most domains, with some concerns related to participant blinding, which is difficult to achieve in studies comparing delivery modes (Appendices 2-4). Overall, the evidence base was considered to be of moderate-to-high methodological quality, supporting the credibility of the review findings.

Patient Satisfaction

Across the 13 included studies, overall maternal satisfaction was generally high regardless of delivery mode. However, most studies reported higher satisfaction among women who experienced vaginal delivery than among those undergoing C-section [2,4-6,14,16]. Karoni et al. [4] reported satisfaction rates of 65.6% (95% CI: 56.97-74.22) for vaginal delivery and 57.2% (95% CI: 48.19-66.20) for cesarean delivery. Similarly, Mohammed et al. [17] found that 93.3% of women who underwent VBAC reported satisfaction compared with 43.3% among women who underwent a C-section after a previous vaginal birth (p<0.001). Dunn et al. [14] reported that women who experienced VBAC felt more prepared for childbirth and experienced less discomfort following delivery than women who underwent C-section. In contrast, Aydin et al. [7] reported significantly higher satisfaction among women who underwent C-section (81.3%) than among those who experienced vaginal birth (70.4%) (p=0.009). Studies by Costa et al. [18] and Yilmaz et al. [10] found no significant differences in satisfaction between delivery modes.

Factors Influencing the Satisfaction

Several factors, including the mother’s education level, domicile (residence areas), planned delivery care, healthcare professional gender, complications, partners’ education levels and employment, pain control measures, Apgar scores, and injuries, had a substantial impact on satisfaction for both types of deliveries [1,4-8,15]. There were no significant differences in satisfaction levels across maternal demographic groups or pregnancy-related characteristics. However, certain features, such as planned pregnancy and excellent prenatal care, improved satisfaction with both vaginal and cesarean deliveries [4,8,10]. The presence of a supporting companion during birth significantly boosted satisfaction levels, especially in primary-care settings [7]. Furthermore, satisfaction with the consent procedure was significantly (p<0.001) associated with the delivery method, with elective and emergency C-sections receiving higher satisfaction ratings in association with informed consent [6].

We found that emergency C-sections and C-sections with inadequate communication are associated with decreased maternal satisfaction and poor outcomes, respectively [15,16]. Porter et al. [15] found that 81% of women who had C-sections were satisfied with their experience. However, 42% of these mothers reported at least one source of distress, such as inadequate communication with healthcare personnel or missing the birth of their child. Aydin et al. [7] reported that women whose spouses had a high level of education, worked themselves, and did not get enemas during labor had higher levels of satisfaction with the C-section. Only one study by Yilmaz et al. [10] found no significant difference in satisfaction scores between women who had vaginal deliveries and those who had cesareans. Complications, such as postpartum hemorrhage, were also associated with dissatisfaction with either delivery mode [5]. Women judged vaginal delivery to be beneficial to their physical and emotional health, whereas the C-section was pain-free but was also considered by women to increase the risk of future complications [2]. 

Discussions

Childbirth is a critical moment for women, affecting their physical, emotional, and psychological well-being [1,2]. Patient satisfaction with delivery care is a critical component in determining the quality of maternal healthcare services [6,19]. This systematic review explored the patient satisfaction between vaginal delivery and C-section and the factors influencing satisfaction levels. Importantly, satisfaction differed substantially between elective and emergency C-sections. Schindl et al. [16] demonstrated that elective C-section was associated with a more positive birth experience than emergency C-section. Similarly, Falk et al. [5] found that emergency cesarean delivery was among the strongest predictors of childbirth dissatisfaction. These findings suggest that dissatisfaction may be driven less by cesarean delivery itself and more by the circumstances surrounding the procedure, particularly loss of control, unexpected intervention, complications, and inadequate communication.

The systematic review found that, while the majority of women reported being satisfied with their delivery experience, there were significant disparities in satisfaction levels depending on the delivery modes (vaginal vs. C-section), aligning with a previous integrative review with 26 studies [20]. Vaginal delivery has generally been associated with higher satisfaction levels than C-sections. Most studies in this review confirmed this, with the vast majority of women indicating a preference for a vaginal birth if given the option again. However, a few studies found that some women who had C-sections had equal or higher levels of satisfaction or did not report any association. This disparity highlights the complexities of patient satisfaction influenced by individual experiences and preferences during childbirth and other multiple factors [9,21,22].

Several factors were found to influence patient satisfaction with both vaginal delivery and C-section. These included maternal education, domicile, intended delivery care services, healthcare professional gender, partner's education and work status, pain control strategies, Apgar scores, and delivery-related injuries. Similar factors were also found to be associated with satisfaction with obstetric care by previous studies [23-25]. We also found that planned C-sections are associated with higher satisfaction than unplanned ones, consistent with what the previous review showed [20]. This is most likely due to the psychological influence of being prepped for the surgical operation in advance. Unplanned cesareans, which are frequently caused by problems/complications during labor, can be stressful and leave you feeling out of control of the birthing process. Our findings also confirm the impact of stress by showing that dissatisfaction was attributed to physical stress and insufficient analgesia. Moreover, previous studies also showed that emergency C-section, feeling ignored and disempowered, loss of control, being uninformed, and loss of birth values that favor vaginal birth contributed to dissatisfaction [19,20], indicating that a woman's emotional state during childbirth significantly impacts her satisfaction. 

The presence of a supporting companion (partner/spouse or family) during birth has emerged as a predictor of satisfaction, especially in primary care settings. This aligns with what previous research has reported, which is that having a companion increased overall satisfaction scores by 5-6%, depending on the healthcare level. Mocumbi et al. [3] found that mothers who experienced bad experiences during the maternal care process, such as abandonment when they needed support, contempt, humiliation, or physical abuse, reported lower levels of satisfaction than those who did not (68.5% vs. 93.5%). Furthermore, they expressed higher levels of discontent (20.1% versus 2.1%). Mothers who gave birth in primary-level facilities were more satisfied than those who gave birth in hospitals. Having a companion increased the overall satisfaction score by 0.06 in type II health centers (CI 0.03-0.10) and by 0.05 in type I health centers (CI -0.02 - 0.13), compared to -0.01 (CI -0.08 - 0.07) in hospitals, irrespective of delivery modes [19]. This highlights the importance of emotional support and companionship throughout labor and delivery, as well as comprehensive maternity care that addresses both physical and emotional well-being. Previous studies showed that most women preferred to have a partner during childbirth to cater to their needs, and healthcare providers perceived the companions as helpful, especially with non-clinical activities and offering emotional support to women [26,27]. 

We also found that specific events during vaginal delivery impacted the satisfaction levels, despite overall high satisfaction with vaginal delivery. Operative vaginal delivery was associated with lower satisfaction, aligning with previous research showing that instrumental vaginal delivery is associated with lower satisfaction than normal vaginal delivery and C-section due to traumatic experiences faced by women [28].

The consent procedure was shown to be associated with satisfaction in conjunction with informed consent in addition to effective communication and involvement of mothers in decision-making processes, aligning with previous studies that showed a significant association between patient satisfaction and factors such as patient-doctor relationship, communication, and other patient-centered care components [20,29,30]. Our review showed that inadequate communication with healthcare practitioners was a major source of distress for women, particularly those undergoing C-sections. Poor communication was associated with stress, uncertainty, and dissatisfaction with the delivery experience, highlighting the necessity of clear and empathic communication between healthcare personnel and their patients. Effective communication and informed consent emerged as important determinants of maternal satisfaction across delivery modes. Our review demonstrated that women who reported better communication with healthcare providers and greater involvement in decision-making experienced higher levels of satisfaction with childbirth. These findings are particularly relevant for women undergoing C-sections, as the procedure involves major abdominal surgery and carries potential risks, including hemorrhage, infection, hysterectomy, and, in rare cases, maternal mortality [22,31]. Adequate counseling regarding the indications, benefits, risks, and expected recovery associated with cesarean delivery may help women develop realistic expectations and maintain a sense of control during childbirth. Consistent with this interpretation, our review found that complications such as postpartum hemorrhage and emergency C-section were associated with lower satisfaction levels [5]. Collectively, these findings highlight the importance of shared decision-making, effective communication, and comprehensive counseling in improving maternal satisfaction regardless of delivery mode.

Interestingly, most demographic and pregnancy-related characteristics were not consistently associated with maternal satisfaction, with the exception of factors such as maternal education in some studies. This suggests that satisfaction may be influenced more strongly by women's experiences of care than by their baseline characteristics. The included qualitative studies revealed notable differences in how women perceived delivery modes. Vaginal delivery was commonly viewed as a natural process that promotes physical recovery, maternal bonding, and emotional well-being, whereas C-section was often perceived as less painful during childbirth but associated with surgical risks and longer recovery periods [2]. Nevertheless, women who underwent cesarean delivery for medical indications frequently reported satisfaction when positive maternal and neonatal outcomes were achieved, indicating that perceptions of safety and clinical necessity may shape satisfaction independently of delivery mode. These findings emphasize the importance of providing individualized education and counseling on the risks, benefits, and recovery expectations associated with different delivery options, thereby enabling women to make informed decisions and fostering a more positive childbirth experience. Open and honest communication with healthcare practitioners about their preferences, medical history, and concerns is essential, and this collaborative approach enables women to make informed decisions based on their specific needs and expectations, resulting in high satisfaction with the delivery experience [20,32].

This review has some limitations. First, screening, study selection, data extraction, and quality assessment were conducted by a single reviewer, which may have introduced selection and extraction bias. Second, substantial heterogeneity existed among the included studies with respect to study design, settings, populations, delivery categories, and satisfaction measurement tools, precluding quantitative meta-analysis. Third, only English-language studies were included, which may have resulted in language bias.

## Conclusions

This systematic review found that maternal satisfaction with childbirth is influenced by multiple clinical, psychosocial, and healthcare-related factors. Overall, vaginal delivery was associated with higher satisfaction in most studies, although planned elective C-section was also associated with favorable satisfaction outcomes in some settings. Emergency C-section, delivery complications, inadequate communication, poor informed consent processes, and reduced participation in decision-making were consistently associated with lower satisfaction. Healthcare providers should prioritize respectful maternity care, effective communication, shared decision-making, emotional support, and adequate pain management to improve women's childbirth experiences, regardless of delivery mode.

## Appendices

Appendix 1

**Table 2:** Database search strategies

Database	Search Strategy	Records Retrieved
PubMed/MEDLINE	("Patient Satisfaction"[Mesh] OR "patient satisfaction" OR "maternal satisfaction" OR "birth satisfaction" OR "childbirth satisfaction" OR "birth experience") AND ("Cesarean Section"[Mesh] OR cesarean section OR caesarean section OR C-section) AND ("Vaginal Delivery"[Mesh] OR vaginal delivery OR vaginal birth OR normal delivery OR spontaneous vaginal delivery)	n = 78
EMBASE	('patient satisfaction'/exp OR 'maternal satisfaction' OR 'birth satisfaction' OR 'birth experience') AND ('cesarean section'/exp OR 'caesarean section' OR 'c-section') AND ('vaginal delivery'/exp OR 'vaginal birth' OR 'normal delivery')	n = 91
Scopus	TITLE-ABS-KEY ("patient satisfaction" OR "maternal satisfaction" OR "birth satisfaction" OR "childbirth satisfaction" OR "birth experience") AND TITLE-ABS-KEY ("cesarean section" OR "caesarean section" OR "c-section") AND TITLE-ABS-KEY ("vaginal delivery" OR "vaginal birth" OR "normal delivery")	n = 58
Web of Science Core Collection	TS=("patient satisfaction" OR "maternal satisfaction" OR "birth satisfaction" OR "childbirth satisfaction" OR "birth experience") AND TS=("cesarean section" OR "caesarean section") AND TS=("vaginal delivery" OR "vaginal birth" OR "normal delivery")	n = 69
Cochrane Library	("patient satisfaction" OR "maternal satisfaction" OR "birth experience") AND ("cesarean section" OR "caesarean section") AND ("vaginal delivery" OR "vaginal birth")	n = 18
Google Scholar	allintitle: ("maternal satisfaction" OR "patient satisfaction" OR "birth experience") ("cesarean section" OR "caesarean section") ("vaginal delivery" OR "vaginal birth")	n =68

Appendix 2

**Table 3:** Quality assessment of observational studies using the Newcastle-Ottawa Scale (NOS) NOS interpretation: 0-3 low quality; 4-6 moderate quality; 7-9 high quality

Study	Selection (4 stars)	Comparability (2 stars)	Outcome/Exposure (3 stars)	Total Score	Quality Category	Main Methodological Concerns
Karoni et al. [4], 2020	★★★★	★★	★★★	9/9	High	Minimal risk of bias
Yilmaz et al. [10], 2020	★★★	★	★★★	7/9	High	Single-center setting
Semiha Aydin et al. [7], 2017	★★★	★	★★★	7/9	High	Potential recall bias
Alderdice et al. [8], 2019	★★★★	★★	★★★	9/9	High	None significant
Falk et al. [5], 2019	★★★★	★★	★★★	9/9	High	Registry-based outcome measurement
Costa et al. [18], 2023	★★★★	★★	★★★	9/9	High	Minimal bias
Dunn et al. [14], 2005	★★★	★	★★★	7/9	High	Modest sample size
Mohammed et al. [17], 2015	★★★	★	★★	6/9	Moderate	Limited adjustment for confounders
Levy et al. [6], 2022	★★★★	★★	★★★	9/9	High	Potential recall bias regarding consent discussions

Appendix 3 

**Table 4:** Quality assessment of qualitative studies using the CASP qualitative checklist Main concerns identified: Porter et al., 2007: Limited discussion of researcher reflexivity; Zakerihamidi et al., 2015: Small sample size and cultural specificity; Guittier et al., 2014: Limited transferability beyond study setting.

CASP Domain	Porter et al. [15], 2007	Zakerihamidi et al. [2], 2015	Guittier et al. [1], 2014
Clear statement of aims	Yes	Yes	Yes
Appropriate qualitative methodology	Yes	Yes	Yes
Appropriate research design	Yes	Yes	Yes
Recruitment strategy appropriate	Yes	Yes	Yes
Data collection is adequately described	Yes	Yes	Yes
Researcher–participant relationship considered	Partial	Partial	Partial
Ethical issues addressed	Yes	Yes	Yes
Data analysis sufficiently rigorous	Yes	Yes	Yes
Findings clearly stated	Yes	Yes	Yes
Research value is clearly demonstrated	Yes	Yes	Yes
Overall appraisal	High quality	High quality	High quality

Appendix 4

**Table 5:** Quality assessment of controlled and interventional studies using Cochrane Risk of Bias Tool (Rob 2) *Non-blinding of participants and outcome assessors, unavoidable due to delivery mode

Domain	Schindl et al. [16], 2003
Randomization process	Some concerns*
Deviations from intended interventions	Low risk
Missing outcome data	Low risk
Measurement of outcomes	Low risk
Selection of reported results	Low risk
Overall risk of bias	Some concerns
